# Deficiency in Repair of the Mitochondrial Genome Sensitizes Proliferating Myoblasts to Oxidative Damage

**DOI:** 10.1371/journal.pone.0075201

**Published:** 2013-09-16

**Authors:** Bartosz Szczesny, Gabor Olah, Dillon K. Walker, Elena Volpi, Blake B. Rasmussen, Csaba Szabo, Sankar Mitra

**Affiliations:** 1 Department of Biochemistry and Molecular Biology, University of Texas Medical Branch, Galveston, Texas, United States of America; 2 Department of Nutrition and Metabolism, University of Texas Medical Branch, Galveston, Texas, United States of America; 3 Department of Internal Medicine, University of Texas Medical Branch, Galveston, Texas, United States of America; 4 Department of Anesthesiology, University of Texas Medical Branch, Galveston, Texas, United States of America; Vanderbilt University Medical Center, United States of America

## Abstract

Reactive oxygen species (ROS), generated as a by-product of mitochondrial oxidative phosphorylation, are particularly damaging to the genome of skeletal muscle because of their high oxygen consumption. Proliferating myoblasts play a key role during muscle regeneration by undergoing myogenic differentiation to fuse and restore damaged muscle. This process is severely impaired during aging and in muscular dystrophies. In this study, we investigated the role of oxidatively damaged DNA and its repair in the mitochondrial genome of proliferating skeletal muscle progenitor myoblasts cells and their terminally differentiated product, myotubes. Using the C2C12 cell line as a well-established model for skeletal muscle differentiation, we show that myoblasts are highly sensitive to ROS-mediated DNA damage, particularly in the mitochondrial genome, due to deficiency in 5’ end processing at the DNA strand breaks. Ectopic expression of the mitochondrial-specific 5’ exonuclease, EXOG, a key DNA base excision/single strand break repair (BER/SSBR) enzyme, in myoblasts but not in myotubes, improves the cell’s resistance to oxidative challenge. We linked loss of myoblast viability by activation of apoptosis with deficiency in the repair of the mitochondrial genome. Moreover, the process of myoblast differentiation increases mitochondrial biogenesis and the level of total glutathione. We speculate that our data may provide a mechanistic explanation for depletion of proliferating muscle precursor cells during the development of sarcopenia, and skeletal muscle dystrophies.

## Introduction

The mitochondria generate the majority of the cellular energy via oxidative phosphorylation (OXPHOS) but are also involved in other important cellular functions including cell death signaling [1,2,3], calcium signaling [4,5] and various biosynthetic pathways [6,7,8]. Thus, maintaining mitochondrial homeostasis, including maintaining integrity of the mitochondrial genome, in mammalian cells is critical for survival [9,10]. Mitochondria are a major cellular source of reactive oxygen species (ROS) generated as a by-product of OXPHOS, which continuously challenges the mitochondria and mitochondrial DNA. Oxidants induce a plethora of damaged bases and strand breaks in the genomes. While unrepaired oxidative lesions cause mutations, single-strand breaks (SSBs), particularly in the proliferating cells, have a more profound effect, by causing a collapse of DNA replication forks leading to formation of double strand breaks (DSBs) [11] and by stalling RNA polymerases during transcription [12]. The mutations due to mispairing of oxidized bases in human mitochondrial DNA are 20- to 100-fold higher than those in the nuclear DNA [13,14,15], but their phenotypic threshold is high because of the multiple copies of the mitochondrial genome [16]. We observed earlier that accumulation of unrepaired SSBs in the mitochondrial genome alone decreases the mitochondrial membrane potential, increases ROS generation and decreases oxidative phosphorylation; the result is the triggering of apoptosis [17]. The DNA base excision repair (BER) is the primary DNA repair pathway that maintains integrity of the nuclear and mitochondrial genomes by repairing damage arising from oxidation, alkylation, deamination, depurination/depirimidination and the repair of abasic (AP) sites and SSBs resulting from spontaneous hydrolysis and oxidation, respectively [18,19,20,21]. Briefly, repair of a base lesion is initiated with its excision by a DNA glycosylase to generate an AP site which is cleaved by AP-endonuclease 1 (APE1) in mammalian cells, leaving the 3’ OH group and a nonligatable 5’ deoxyribose phosphate (dRP) residue. This 5’ blocking group is removed by DNA polymerase β (Pol β) or Polγ via their intrinsic dRP lyase activity in the nucleus and mitochondria, respectively [22]. Excision of the oxidized base by DNA glycosylases with intrinsic AP lyase activity generates either 5’ phosphate and the 3’ blocking phospho-ά,β unsaturated aldehyde; or 3’ phosphate, which is subsequently removed by the intrinsic 3’ phosphodiesterase activity of APE1; or 3’ phosphatase activity of PNKP, respectively [23]. Further oxidation of deoxyribose moiety at the terminus of the SSB precludes removal of these lesions by the dRP lyase activity of Polß or Polγ. In this case, the 5’ blocking group in the nuclear DNA together with additional nucleotides are removed by flap endonuclease 1 (FEN1), a 5’ exo/endonuclease. Therefore, the resulting gap filling by a DNA polymerase and nick sealing by a DNA ligase (Lig) during BER can proceed via two subpathways: single-nucleotide (SN)-BER or long-patch (LP)-BER, which remove a single nucleotide or 2-6 additional nucleotides, respectively, at the 5’ terminus [24]. Unlike the situation in the nucleus with multiple DNA polymerases and ligases, their sole mitochondrial counterparts, Polγ and Lig3, are responsible for both replication and repair of mitochondrial DNA [25,26,27,28]. Mammalian mitochondria are proficient in SN-BER [29] and LP-BER, as reported by us [30] and others [31,32]. The presence of several 5’ exonucleases in mammalian mitochondria has been reported, including FEN1 [32], DNA2 [33] and EXOG [34,35]. We recently showed that EXOG provides the major activity to process the 5’ terminus during mitochondrial LP-BER [17].

Skeletal muscle has the unique ability to increase its metabolic rate 100-fold during maximal contractile activity [36]. This increase is associated with increases in the level of oxidative damage, especially in the mitochondria [37]. We previously reported that the skeletal muscle of mice may be particularly susceptible to oxidative stress due to lower nuclear and mitochondrial BER and expression of antioxidant enzymes in comparison to other tissues like liver or kidney [38]. The mitochondrial genome is significantly more sensitive to oxidative damage than its nuclear counterpart [15,39,40], due to its unchromatinized structure, close proximity to OXPHOS and lack of introns [41]. The connection between mitochondrial genome damage and its pathophysiological consequences is being intensively studied in sarcopenia (an age-dependent loss of mass and strength of the skeletal muscle) and during various forms of muscular dystrophy [42,43,44,45,46,47,48,49]. Although apoptosis plays an important role in skeletal muscle development and regeneration by controlling the population of proliferating myoblasts [50,51,52], the pathophysiological link of oxidative stress and the role of the integrity of the nuclear and mitochondrial genomes during myogenic differentiation is unknown.

In this study, we investigate, for a first time, the effect of oxidative DNA damage, particularly SSBs and their repair, in proliferating myoblasts and terminally differentiated myotubes, using mouse C2C12 cells as a well-established cellular model of skeletal muscle differentiation [53,54,55]. We hypothesized that proliferating myoblasts are highly sensitive to oxidative damage due to a deficiency in the repair of the mitochondrial genome, which, in turn causes a loss of their viability by activation of the apoptotic pathway.

## Material and Methods

### 2.1: Reagents

Reverse-phase HPLC-purified oligonucleotides were purchased from the Midland Certified Reagent Company (Midland, TX); their sequences are shown in Table **1**. We obtained [γ-^32^P] ATP (3000Ci/mmol), [ά-^32^P] dCTP (3000Ci/mmol) and [ά-^32^P] dATP (3000Ci/mmol) from PerkinElmer Inc. (Waltham, MA); glucose oxidase (GOx) from *Aspergillus niger* (180,000U/mg) was obtained from Sigma-Aldrich (St. Louis, MO). All cell culture media and supplements were purchased from Invitrogen Life Technologies (Carlsbad, CA).

**Table 1:** Oligonucleotides used in this study.

Oligo1:
5' GAT CTG ATT CCC CAT CTC CTC AGT TTC ACT **U** CTG CAC CGC ATG 3'
3' CTA GAC TAA GGG GTA GAG GAG TCA AAG TGA **G** GAC GTG GCG TAC 5
Oligo2:
5'-GAT CTG ATT CCC CAT CTC CTC AGT T **5OHU** TC ACT AGT GAA GGC ATG CAC CCT TCT-3’
3'-CTA GAC TAA GGG GTA GAG GAG TCA A **G** AG TGA TCA CTT CCG TAC GTG GGA AGA-5'
Oligo3:
5'-GAT CTG ATT CCC CAT CTC CTC AGT TTC ACT **THF** AGT GAA GGC ATG CAC CCT TCT-3’
3'-CTA GAC TAA GGG GTA GAG GAG TCA AAG TGA **C** TCA CTT CCG TAC GTG GGA AGA-5'
Ologo4:
5’-GCT TAGCTT GGA ATC GTA TCA TGT ACA CTC G
5’ -CGA ATCGAA CCT TAG CAT AGT ACA TGT GAG CAC ACG GCA CAT CTG GCA CGG-5’
Oligo5:
5’-GCT TAGCTT GGA ATC GTA TCA TGT A CACTC GTG TGC CGT GTA GAC CGT GCC-3’
5’ -CGA ATCGAA CCT TAG CAT AGT ACA T GTGAG CAC ACG GCA CAT CTG GCA CGG-5’

### 2.2: Cell culture

The mouse muscle myoblasts cell line (C2C12) was obtained from the ATCC (cat# CRL-1772). Undifferentiated, proliferative active myoblasts were cultured in DMEM containing 10% heat-inactivated fetal bovine and differentiation was induced by changing the culture medium to DMEM containing 2% horse serum. Both media were supplemented with 50 units/mL penicillin and 50 µg/mL streptomycin; and cell cultures were maintained at 37°C, 5% CO_2_.

### 2.3: Measurement of ROS

Myoblasts or myotubes cultured in 24-well plates were washed in serum-free media and incubated for 20 min at room temperature with 25µM of 2',7'-dichlorodihydrofluorescein diacetate (H_2_DCFDA) (Molecular Probes cat#D399). Cells were washed twice with serum-free media and incubated with media containing three different concentration of GOx (0.005, 0.05 and 0.5 U/ml). The change in fluorescent signal from nonfluorescent H_2_DCFDA to the highly fluorescent 2',7'-dichlorofluorescein (DCF) was monitored by Microplate Fluorescence Reader (Bio-Tek Instruments Inc., Winooski, VT) at 0, 5, 15, 30 and 60 min after GOx was added.

### 2.4: Preparation of whole cell, nuclear and mitochondrial extract; and Western analysis

Whole cell, nuclear and mitochondrial extracts were prepared as described earlier [38]. The protein concentration was determined with Bradford reagent (Bio-Rad, Hercules, CA) using bovine serum albumin (BSA) as the standard. Western analysis was performed as described earlier [56] with the membranes sequentially probed using antibodies against: DNA Ligase 3 (QED Bioscience Inc., San Diego, CA), Pax7 (R&D Systems, Minneapolis, MN), PCNA and myogenin (Santa Cruz Biotechnology, Santa Cruz, CA), nucleolin (Abcam, Cambridge, UK), APE1 (Novus Biologicals, Littleton, CO), GAPDH, caspase-9, caspase-8 and caspase-3 (Cell Signaling Technology, Danvers, MA), the 56KDa subunit of ATP synthase (Molecular Probe-Invitrogen, Carlsbad, CA) and FLAG-HRP conjugated (SIGMA, St. Louis, MO).

### 2.5: Myoblasts transfection with EXOG or OGG1

Human EXOG cDNA was cloned into p3x-FLAG-CMV-14 vector (SIGMA, St. Louis, MO) as described earlier [34,35]. Human cDNA of mitochondrial-specific OGG1 was cloned in pCDEB mammalian expression plasmid [57]. The myoblasts were transfected using Lipofectamine 2000 (Invitrogen, Carlsbad, CA) per the manufacturer’s protocol. At 24 h after myoblast transfection, differentiation was initiated by media change. Expression of EXOG was monitored by Western analysis with anti-FLAG-specific antibodies.

### 2.6: Quantification of DNA damage

Total DNA from mock- and GOx-treated cells was isolated using DNase Blood and Tissue Kit (QIAGEN, Hilden, Germany). Gene-specific semi-quantitative PCR assays to measure DNA damage were performed as described earlier [58] using LongAmp Taq DNA Polymerase (New England BioLabs, Ipswich, MA). Briefly, damage to nuclear DNA was estimated by quantification of the PCR amplification of the 9kb nuclear-specific DNA fragment using PicoGreen **fluorescent dye to detect double-stranded DNA (Quant-iT™ PicoGreen, Molecular Probe**). Damage to the mitochondrial DNA was estimated by quantification of the PCR amplification of the 10kb mitochondrial-specific DNA fragment using PicoGreen staining. Obtained data was normalized by the PCR amplification of 117bp mitochondrial genome-specific fragment for correction of the multiple copies of the mitochondrial genome. Preliminary assays were carried out to ensure the linearity of PCR amplification with respect to the number of cycles and DNA concentration.

### 2.7: Assay of BER

The total repair assay was carried out as described earlier [17,30,59]. The sequences of oligonucleotide duplexes used in this study are shown in Table 1. Repair of uracil- (U), 5-hydroxyuracil- (5-OHU) or tetrahydrofuran- (THF) containing oligo duplexes were assayed in 20 µL assay buffer containing 20 µM each of 4 unlabeled dNTPs, 4 µCi [ά-^32^P] dCTP or [ά-^32^P] dATP, and 5 µg of the nuclear or mitochondrial protein extract. After incubation at 37°C for 60 min, the reaction was terminated by addition of 10 µl of 70% formamide. The APE-specific activity was determined as described before [60] with oligo3 (Table 1) labeled at the 5’-end with [γ-^32^P] ATP and T4 polynucleotide kinase (New England BioLabs, Ipswich, MA) and annealed with an equimolar amount of the complementary strand with C opposite the THF residue. The 20 µl reaction was carried out in 50 mM Tris-HCl (pH 8.6), 50 mM KCl, 2 mM MgCl_2_, 0.2 pmol ^32^P-labeled oligo and 50 nM unlabeled oligo substrate. After incubation with 30 ng nuclear extracts isolated from myoblasts or myotubes at 37°C for 10 min, 10 µl loading solution (90% formamide, 0.05% bromophenol blue and 0.05% xylene cyanol) was added to the assay mixture to terminate reaction. DNA polymerase and ligase activity was assayed as described earlier [61,62]. Activity of DNA polymerases was measured using oligo4 (Table 1) and 1 µg of the nuclear extract isolated from the myoblasts or the myotubes, for 30 min at 37°C. Incorporation of nucleotides was monitored with the presence of [ά-^32^P] dATP in the reaction mixture. The DNA ligation was assayed using oligo5 duplex (Table 1) with 26-mer 5’-end labeled using [γ-^32^P] ATP and T4 polynucleotide kinase (New England BioLabs, Ipswich, MA) before annealing, for 1 h at 37°C. In analysis of all BER activities, the product was separated from the substrate by electrophoresis in a 20% acrylamide/7M urea gel. The radioactivity in substrate/product bands was quantitated by PhosphorImager (Molecular Dynamics, Sunnyvale, CA) using ImageQuant software. Preliminary enzyme assays were carried out to ensure the linearity of the reaction with respect to both time and the amount of extracts.

### 2.8: Activity of citrate synthase

The specific activity of citrate synthase was analyzed using a Citrate Synthase Assay Kit (SIGMA-Aldrich, St. Louis, MO) according to manufacturer’s recommendations, to calculate the mitochondrial volume of myoblasts and myotubes, as described before [63].

### 2.9: MTT cell viability assay

Viability of the mock- and GOx-treated myoblasts and myotubes was determined using a Cell Proliferation (MTT) kit (Roche, Applied Science, Penzberg, Germany) according to manufacturer’s recommendation. Briefly, ~7,000 cells were seeded into each well of a 96-well plate, with ~50% or 90% confluency for the myoblasts and myotubes, respectively. Myoblasts were differentiated into myotubes after incubation for 4 days in differentiated media. Cells were treated with various concentration of GOx for 1 h, and 24 h later loaded with 10 mg/ml of MTT-labeling reagent. The cells were incubated for additional 4 h, followed by the addition of the solubilization solution and incubation overnight at 37°C. The changes in absorbance were monitored using a micro-plate reader (Bio-Rad, Hercules, CA) at 570 nm. The absorbance of control, untreated cells was considered to indicate 100% cell viability. To test the effect of caspase inhibitors on the viability of myoblasts and myotubes treated with GOx, cells were pre-incubated for 30 min with 10 or 30µM Z-VAD-FMK (BD, Bioscience), the general caspase inhibitor, prior to the GOx challenge.

### 2.10: Total Glutathione (GSSG+GSH) measurement

The content of total glutathione in the myoblasts and myotubes was determine using OxiSelect Total Glutathione (GSSG/GSH) assay kit (Cell Biolabs, San Diego, CA) according to manufacture’s recommendation.

### 2.11: Annexin V- propidium iodide (PI) staining for apoptosis/necrosis detection by flow cytometry

The detection of apoptosis/necrosis of myoblasts treated with 0.005 and 0.02 U/ml of GOx was performed 4 h after treatment by Annexin V-PI staining followed by flow cytometry using Annexin V-FITC Apoptosis Detection Kit according to manufacture’s recommendation (Sigma-Aldrich, St. Louis, MO). Briefly, control and treated cells were trypsinized, washed in ice-cold PBS and re-suspended in 1 ml Binding Buffer. Then, 1×10^5^ cells (500 µl) were incubated with 5 µl Annexin V FITC and 10 µl PI for 10 min at 25°C in the dark, and analyzed immediately using a Guava EasyCyte Plus Flow Cytometer (Millipore, Billerica, MA). The early and late apoptotic cells, and as well as the necrotic cells, were estimated as the percentage of the total number of cells by CytoSoft 5.3 Software (Millipore, Billerica, MA).

### 2.12: Statistical analysis

At least three independent experiments were carried out in duplicate for each assay. The results are presented as mean ± standard error (s.e.m.) and analyzed for statistical significance with one-way ANOVA. A *p* value ≤ 0.05 was considered as statistical significant and marked by *.

## Results and Discussion

### 3.1: Differentiated myotubes have higher mitochondrial volume and total glutathione level

**Figure 1:**
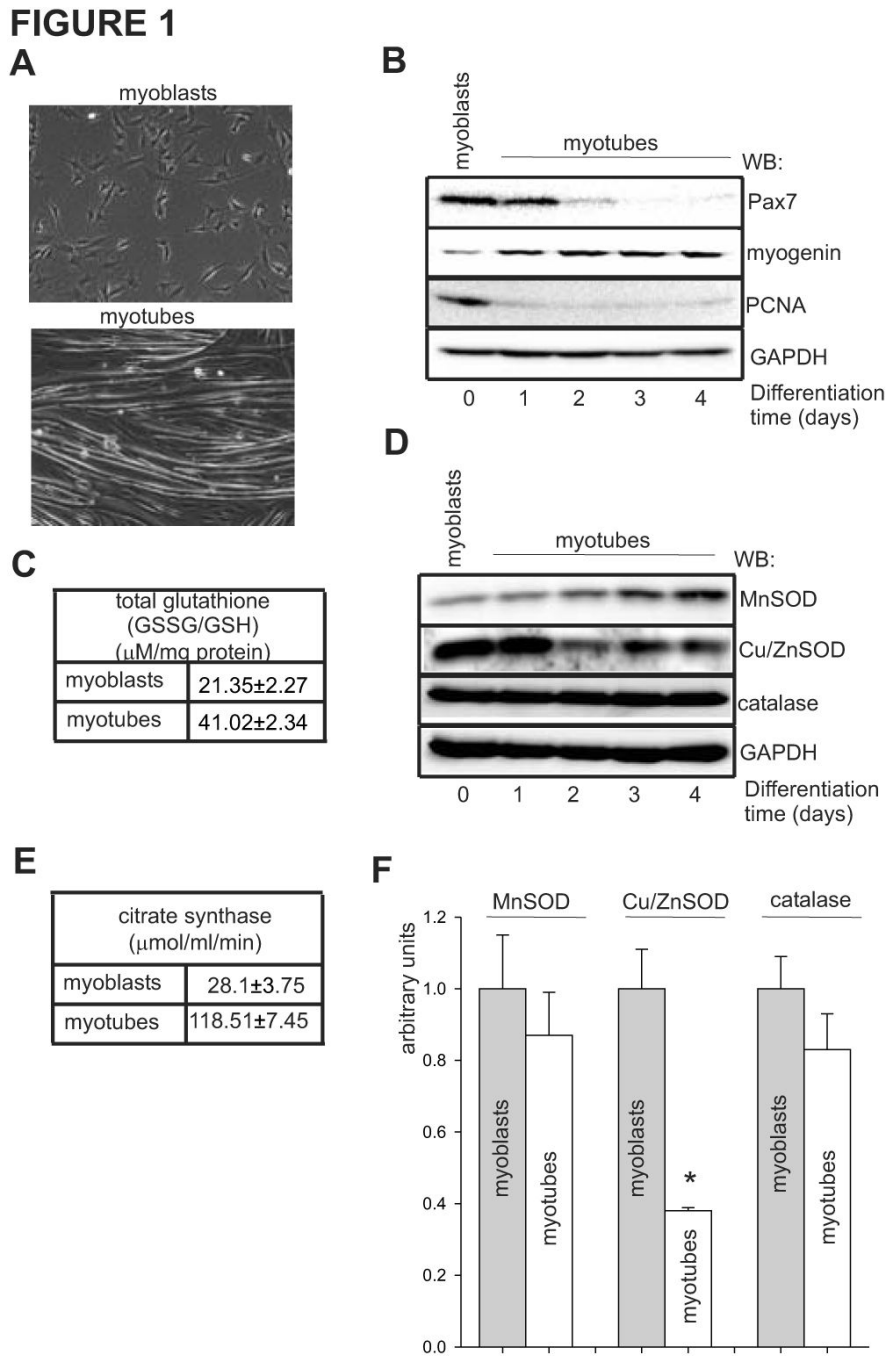
Differentiated myotubes have higher mitochondrial volume and elevated total glutathione level. (**A**) Morphological differences between undifferentiated myoblasts and terminally differentiated, fused myotubes shown by phase-contrast microscopy. (**B**) The effect of myoblast differentiation on expression levels of Pax7, myogenin and PCNA (differentiation markers) monitored on days 0-4 by Western analysis of total cell extracts. The level of GAPDH was used as a loading control. (**C**) The level of total glutathione (GSSG+GSH) in total cell extracts of control myoblasts and myotubes expressed as µM/mg protein. (**D**) Changes in expression level of MnSOD, Cu/ZnSOD and catalase as a function of myoblasts differentiation. The level of GAPDH was used as a loading control. (**E**) The effect of myoblasts differentiation on mitochondrial biogenesis, as calculated by specific activity of citrate synthase localized exclusively in the mitochondria. (**F**) Quantification of normalized levels of MnSOD (to the citrate synthase activity), and Cu/ZnSOD and catalase (to the expression level of GAPDH) in myoblasts and myotubes based on analysis of three independent Western blots. * indicates *p* ≤ 0.05 relative to myoblasts.

To test the importance of the integrity of the nuclear and mitochondrial genomes and their faithful repair during skeletal muscle differentiation, we used mouse C2C12 cells. The C2C12 cellular model recapitulates the process of muscle cells differentiation *in vivo*, as shown by irreversible cell cycle withdrawal, repression of cell proliferation-associated genes and expression of terminally differentiated muscle-specific genes [64]. Proliferating myoblasts differ morphologically from terminally differentiated, non-proliferating myotubes (Figure 1A), as they express different sets of proteins. To confirm proper differentiation, we monitored the expression of the transcription factor paired-box 7 (Pax7) and proliferating cell nuclear antigen (PCNA), known to be inhibited during myoblast differentiation or by repression of cellular proliferation, respectively [65,66], as well as expression of myogenin, known to be positively associated with differentiation [67]; these were accomplished by Western analysis of myoblasts at days 0-4 after induction of differentiation. The results clearly indicate that the process of myoblast differentiation is accurately recapitulated, as shown by decreased expression of Pax7 and PCNA and increased expression of myogenin (Figure 1B). In all subsequent experiments, we used myoblasts and terminally differentiated myotubes at 4 days after induction of differentiation.

Cellular protection against oxidative damage includes elimination of ROS by antioxidant molecules (e.g., glutathione), ROS-inactivating antioxidant enzymes, removal of damaged macromolecules and the repair of oxidatively damaged DNA. First, we calculated the total level of glutathione (GSSG+GSH) in myoblasts and myotubes. We detected a 2-fold increase in the level of total glutathione in myotubes in comparison to myoblasts, 21.3±2.2 and 42.0±2.3 µM/mg protein, respectively (Figure 1C), similarly as reported before [68,69,70]. Next, we compared the expression of three major antioxidant enzymes, namely MnSOD, CU/ZnSOD and catalase, by Western blot analysis (Figure 1D). Due to their different subcellular localizations, we normalized the level of MnSOD to the specific activity of citrate synthase and the level of Cu/ZnSOD and catalase to the expression level of GAPDH, since these are localized in the mitochondria and cytoplasm, respectively. Similar to earlier reports [55], we observed a 4-fold increase in citrate synthase activity in the myotubes compared to myoblasts (Figure 1E), indicating an increase in mitochondrial biogenesis as a function of myoblasts differentiation. Although we observed increased expression of MnSOD during differentiation in the total cell extract, the normalized level of MnSOD was slightly reduced in the myotubes, as compared to myoblasts (Figure 1D, F). The normalized expression of Cu/ZnSOD was reduced by 70% in differentiated myotubes, while catalase levels remained essentially unchanged (Figure 1F). Together, obtained data showed that the process of myoblast differentiation increases mitochondrial biogenesis and the level of total glutathione, while negatively regulating the expression of Cu/ZnSOD.

### 3.2: Mitochondrial genome of proliferating myoblasts is highly susceptible to oxidative damage

**Figure 2:**
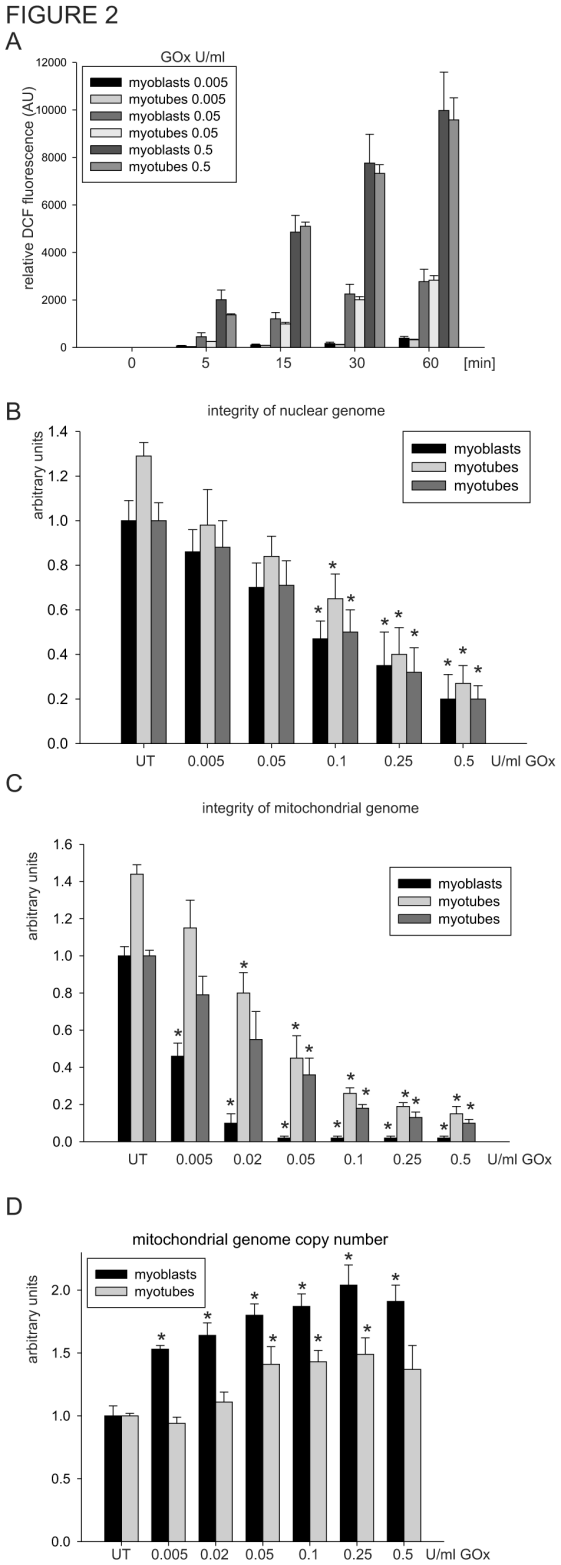
Mitochondrial genome of myoblasts is highly sensitive to oxidant-induced damage. (**A**) The amount of ROS generated by 0.005, 0.05 and 0.5 U/ml of GOx detected by fluorescence of DCF at 0, 5, 15, 30 and 60 min in cultured myoblasts and myotubes. Integrity of the nuclear (**B**) and mitochondrial (**C**) genomes of myoblasts and myotubes cultured for 1 h in various concentrations of GOx analyzed by amplification of 9kb and 10kb of nuclear- and mitochondrial-specific DNA fragments, respectively, by long-amplicon (LA)-PCR technique. (**D**) Mitochondrial genome copy number was analyzed by amplification of the 117bp mitochondrial genome-specific DNA fragment. The mitochondrial genome copy number in UT myoblasts and myotubes were set as 1. The graphs are based on PCR amplification with three independently isolated DNA for each experimental point and shown as mean ± standard error (s.e.m.). The integrity in untreated (UT) control myoblasts was set as 1. The integrity in UT myotubes is shown relative to UT myoblasts or set as 1. Myotubes at day 4 of differentiation were used. * indicates *p* ≤ 0.05 relative to UT controls.

Next, we examined the effect of ROS on the integrity of both the nuclear and mitochondrial genomes, by challenging myoblasts and myotubes with various concentrations of glucose oxidase (GOx), which in the presence of glucose generates hydrogen peroxide [71]. To verify that GOx induces the production of similar amounts of hydrogen peroxide in both cells, we monitored the fluorescence of oxidized DCF dye. As shown in Figure 2A, a similar amount of ROS with three different concentration of GOx was detected for both cells in a time-dependent manner. Hence, we treated myoblasts and myotubes with various concentrations of GOx for 1 h and measured the integrity of their genomes by PCR of long DNA targets. This method allows us to monitor directly the integrity of both the nuclear and mitochondrial genome without separate isolation of the nuclear and mitochondrial DNA [58]; and detects mostly SSBs because the majority of oxidative DNA base lesions are by-passed by replicative DNA polymerases [72,73]. To facilitate comparisons, we set the amount of PCR amplification with nuclear- and mitochondrial-specific primers in untreated (UT) myoblasts at 1 and the obtained PCR signal in myotubes was quantified in two separate ways: i) relatively to the myoblasts and ii) independently, when it was also set as 1. This approach allowed us to monitor the difference between myoblasts and myotubes as well as evaluate both cell types independently. As expected, amplification of the 9kb nuclear genome-specific DNA fragment decreased with increasing concentrations of GOx, in both myoblasts and myotubes (Figure 2B). Although we observed ~30% higher integrity of the nuclear genome in UT myotubes compared to the myoblasts, the sensitivity to oxidative damage of the nuclear genome in both cell types was comparable (Figure 2B). Similarly, the integrity of the mitochondrial genome in UT myotubes was higher than in control myoblasts (Figure 2C), but the mitochondrial genome of myoblasts was much more sensitive to hydrogen peroxide than that of the myotubes (Figure 2C). Even at the relatively low GOx concentration (0.02U/ml), less than 10% of the mitochondrial genome was intact in myoblasts compared to 60% of myotubes (Figure 2C). Under similar conditions, more than 90% of the nuclear genome in both cells remained unaffected (Figure 2B). It is generally accepted that the mitochondrial genome is more sensitive to oxidative damage than its nuclear counterpart, mainly due to its unchromatinized nature and close proximity to OXPHOS [13,14,15], and our data support this assumption (Figure 2B,C). However, the observed difference in the sensitivity of the mitochondrial genome to oxidative damage between the two cell types was unexpected. We speculated that, since comparable levels of ROS were generated by GOx in myoblasts and myotubes (Figure 2A), the observed difference in oxidative damage of the mitochondrial genome of myoblasts must be due to intrinsic properties of these two respective cell types. The replication of the mitochondrial genome is cell cycle independent [74]; thus, we analyzed changes in the mitochondrial DNA copy number of the myoblasts and myotubes and as a function of increasing concentrations of hydrogen peroxide. Interestingly, although we detected 3-fold more mitochondrial genomes in the myotubes, which correlates with increases in mitochondrial biogenesis (Figure 1E), we observed a steady increase in the number of mitochondrial genome copies in both cells as a function of increasing concentration of GOx, with slightly more in the myoblasts (Figure 2D). We speculate that increasing mitochondrial genome replication may be one of the first, rapid-onset mechanisms which serves to compensate for sudden increases in oxidative stress.

It has been reported that proliferating and active myoblasts rely mostly on ATP derived from glycolysis, while in fully differentiated muscle cells, OXPHOS is the predominant source of ATP [75,76]. The bioenergetic remodeling that occurs during skeletal muscle differentiation leads to changes in the mitochondrial ROS synthesis profile [55,77], which may influence the sensitivity of the mitochondrial DNA to oxidative damage. The 16.5kb circular, plasmid-like mitochondrial genome of mammalian cells is organized in a packed nucleoid structure associated with the inner mitochondrial membrane [78,79,80]; whether structural changes in the organization of nucleoids of proliferating vs. terminally differentiated cells may, in turn, reflect an increase vulnerability to oxidative damage remains to be investigated. In addition, oxidants have been shown to have a more profound effect on induction of the mitochondrial genome resulting in mitochondrial dysfunction [81]. To the best of our knowledge, this is the first report showing differences in the sensitivity of the mitochondrial genome to oxidative damage between proliferating and resting cells; it is conceivable that this difference reflects corresponding differences in the DNA repair capacities of different cell states.

### 3.3: The repair capacities for oxidative damage in nucleus of myotubes is highly reduced

**Figure 3:**
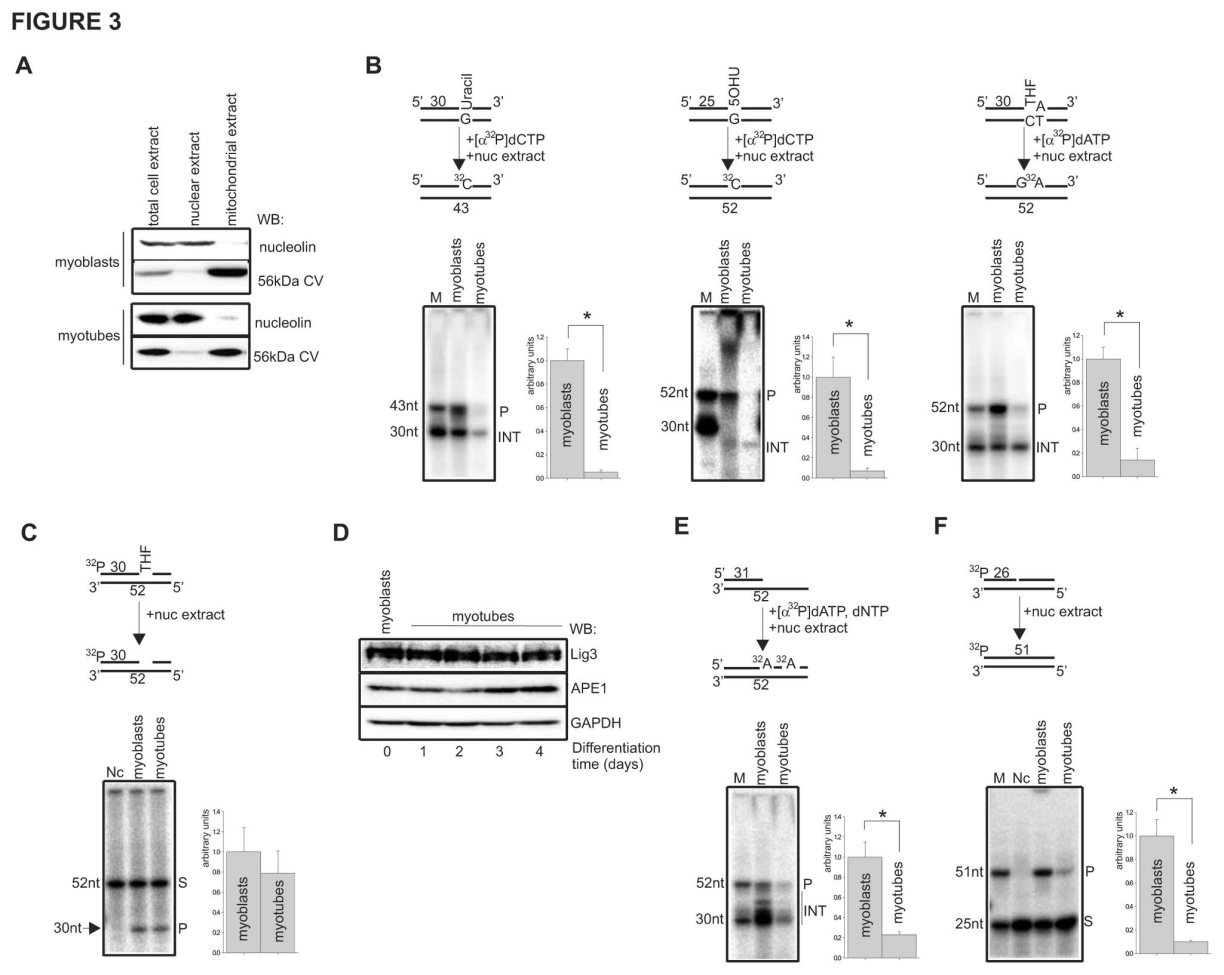
The nuclear extracts of myotubes are deficient in DNA polymerase and DNA ligase activity. (**A**) Purity of the myoblast and myotube nuclear and mitochondrial fractions monitored by Western analysis with nucleolin and the 56kDa subunit of ATP synthase antibodies. (**B**) Total repair synthesis of uracil (U), 5OH-uracil (5OHU) and tetrahydrofuran (THF); (**C**) APE1 endonuclease activity in nuclear extracts of myoblasts and myotubes. (**D**) The effect of myoblast differentiation on expression levels of Lig3 and APE1 (key base excision repair enzymes) monitored on days 0-4 by Western analysis of total cell extracts. The level of GAPDH was used as a loading control. Activities of (**E**) DNA polymerase and (**F**) DNA ligase in nuclear extracts of myoblasts and myotubes. Schematic representation of each repair reaction is shown above the representative radiogram. Relative repair efficiency for each reaction is based on analysis of at least three independently isolated nuclear extracts. Activities in the nuclear extracts of myoblasts were set as 1. Myotubes at day 4 of differentiation were used. * indicate *p* ≤ 0.05. P, final repair product; INT, repair intermediates; S, substrate; M, marker; Nc, negative control.

The major pathway for correcting oxidatively damaged DNA in both the nuclear and the mitochondrial genomes is the BER/SSB repair pathway [18,19,20]. Therefore, we next analyzed the BER activity in nuclear fractions of myobasts and myotubes. Purity of the nuclear and mitochondrial extracts was determined by Western analysis with antibodies against nucleolin and 56kDa subunits of ATP synthase, localized exclusively in the nucleus and the mitochondria, respectively (Figure 3A). Only fractions without significant cross contamination were used in further experiments. First, we tested nuclear extracts of myoblasts and myotubes for total repair efficiency for uracil (U), 5-hydroxy uracil (5-OHU) and tetrahydrofuran (THF) whose repair follows the SN- (U, 5-OHU) and LP-BER (THF) pathway. Generally, the repair efficiency for all tested base lesions were several-fold lower in the nuclear extracts of myotubes compare to myoblasts (Figure 3B). Since, the excision of these lesions from oligo duplexes during repair is initiated by distinct enzymes: uracil DNA glycosylase (UDG), **nei-like 1/2 DNA glycosylase** (**N**EIL1/NEIL2) or endonuclease three homolog 1 DNA glycosylase (NTH1) and apurinic endonuclease 1 (APE1), respectively [20]; our observation strongly suggest general decrease in BER activities in resting myotubes. It was previously reported that nuclear extracts of myotubes are defective in BER activity, due to reduced level of Lig1, resulting in an accumulation of unligated repair intermediates [82]. Since we detected a reduction not only of the final repair product (P) but also in the level of repair intermediates (INT, Figure 3B), particularly for U and 5-OHU, we next examined the individual activities of APE1, UDG, DNA polymerase and DNA ligase to detect which step of the BER process is affected during differentiation of the skeletal muscle cells. Interestingly, comparable levels of the 30nt-long cleavage product of the 52 nucleotide THF-containing oligo in the nuclear extracts of myoblasts and myotubes was observed (Figure 3C), indicating that myoblast differentiation does not affect APE1 activity. This result was further confirmed by Western analysis of the APE1 level (Figure 3D). Similarly, we did not detect any significant differences in activity of UDG in the nuclear extracts of both cells (data not shown). In contrast, we detected a 5-fold reduction in DNA polymerase (Figure 3E) and a 10-fold decrease in DNA ligase activities (Figure 3F) in the nuclear extracts of myotubes, in comparison to myoblasts. Although an earlier report indicated that the level of Polβ involved in nuclear BER was unchanged during muscle cells differentiation [82], the involvement of replicative DNA polymerase δ/ε in the repair of oxidized DNA bases has not yet been investigated. Interestingly, we could not detect changes in the expression level of Lig3 during myoblast differentiation (Figure 3D). Therefore, the decrease in DNA ligase activity observed in the current study is likely the consequence of a decreased expression of Lig1, as previously shown [82]. It is generally accepted that, in the SN-BER, the single nucleotide is replaced by Polß and the gap is rejoined by Lig3, in opposite to the LP-BER, where Polδε together with Lig1 complete the repair [18,83,84,85]. However, the interaction of Polß with Lig1 in nuclear LP-BER has also been reported [86]. Together, our data show a significant deficiency in repair activity for oxidatively damaged DNA bases in the nuclear extracts of myotubes, which is linked with reductions in both DNA repair synthesis and ligation.

### 3.4: Mitochondrial extract of myoblasts accumulates oxidative damage repair intermediates

**Figure 4:**
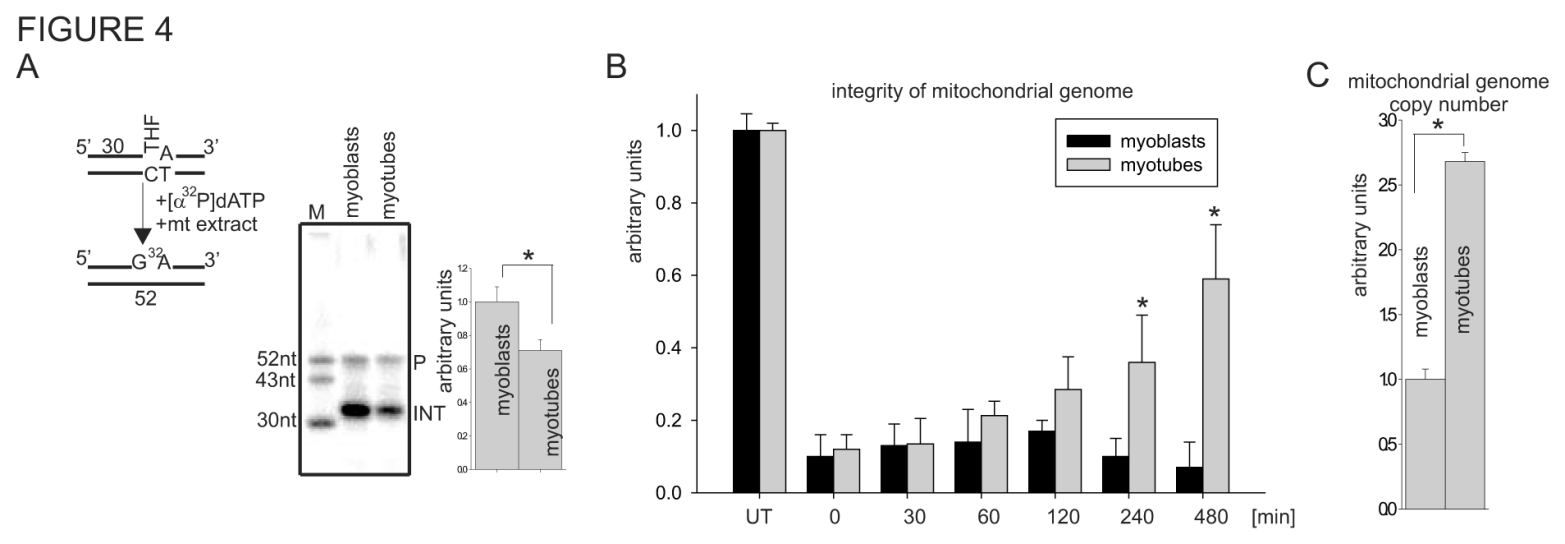
Mitochondrial extracts of myoblasts accumulate DNA repair intermediates. (**A**) Total repair synthesis activity of tetrahydrofuran (THF) containing oligo3 duplex in the mitochondrial extract of myoblasts and myotubes. Schematic representation of repair reaction is shown above the radiogram. Repair efficiency is based on analysis of at least three independently isolated mitochondrial extracts for each cell type. Activity in the mitochondrial extracts of myoblasts was set as 1. * indicates *p* ≤ 0.05. (**B**) Repair efficiency of the mitochondrial genome of myoblasts and myotubes after GOx treatment was monitored by amplification of a 10kb mitochondrial-specific DNA fragment by LA-PCR. The level of integrity in UT myoblasts and myotubes was set as 1. (**C**) The relative number of mitochondrial genomes in myoblasts and myotubes was based on PCR amplification of 117bp mitochondrialDNA-specific fragment. The graphs represent PCR amplification of three independently isolated DNA for each experimental point and shown as mean ± standard error (s.e.m.). Myotubes at day 4 of differentiation were used. P, final repair product; INT, repair intermediates; UT, untreated control.

Recent data indicate that repair of the oxidative damaged mitochondrial genome proceeds mainly through multinucleotide repair patch, a signature of the LP-BER pathway (our unpublished data and [31]). Thus, we analyzed total repair efficiency using THF-containing oligo, which repair proceeds through LP-BER, and the mitochondrial extracts of myoblasts and myotubes. Although the total repair activity of the mitochondrial extract of myotubes was slightly reduced compared to that of myoblasts, we noticed significant accumulation of repair intermediates (INT), particularly in the mitochondrial extracts of myoblasts (Figure 4A). The strong signal observed in this assay represents an incorporation by Polγ radiolabelled nucleotide, ruling out any deficiency in DNA repair synthesis mediated by Polγ, but rather indicating reduced 5’ end-processing or DNA ligation step. However, as we showed earlier, we could not detect changes in the level of Lig3 during myoblast differentiation (Figure 3D), the only DNA ligase present in the mitochondria [27,87]. Thus, we speculate that processing is impaired of the 5’-end at the DNA strand break during repair in the mitochondrial extracts of myoblasts. This scenario could also explain the rapid accumulation of SSBs in the mitochondrial genome of myoblasts after GOx treatment as repair intermediates (Figure 2C). To confirm this hypothesis, we induced a similar level of DNA damage in the mitochondrial genome of the myoblasts and the myotubes, by incubating them for 1 h with 0.02 U/ml and 0.25 U/ml of GOx, respectively. Under these conditions, the integrity of the mitochondrial genome in both cells was reduced to a comparable level (Figure 4B), similar to what we showed earlier (Figure 2C). Next, we monitored the rate of mitochondrial genome repair at various times. A steady increase in mitochondrial genome integrity, indicating a proper repair process, was observed in the myotubes but not the myoblasts, confirming a deficiency in DNA repair of the latter (Figure 4B). We cannot exclude the possibility that the observed difference is related to replication of the mitochondrial genome, which is cell cycle independent [74]. Although we detected a 3-fold higher number of mitochondrial genome in the myotubes in comparison to myoblasts (Figure 4C), replication of the mitochondrial genome could not account for this deficiency in repair of the mitochondrial genome in the myoblasts, since we could not detect major changes in this parameter (Figure 2D). An increased number of mitochondrial genomes in myotubes correlates with an increase of mitochondrial biogenesis, which was observed during the process of myoblast differentiation (Figure 1E). Together, our data indicate that a deficiency in the 5’ end-processing step of mitochondrial BER in myoblasts is the most likely cause of the accumulation of repair intermediates during oxidative stress.

### 3.5: Ectopic expression of EXOG, mitochondrial-specific 5’ endonuclease, increases resistance to oxidative stress of proliferating myoblasts

**Figure 5:**
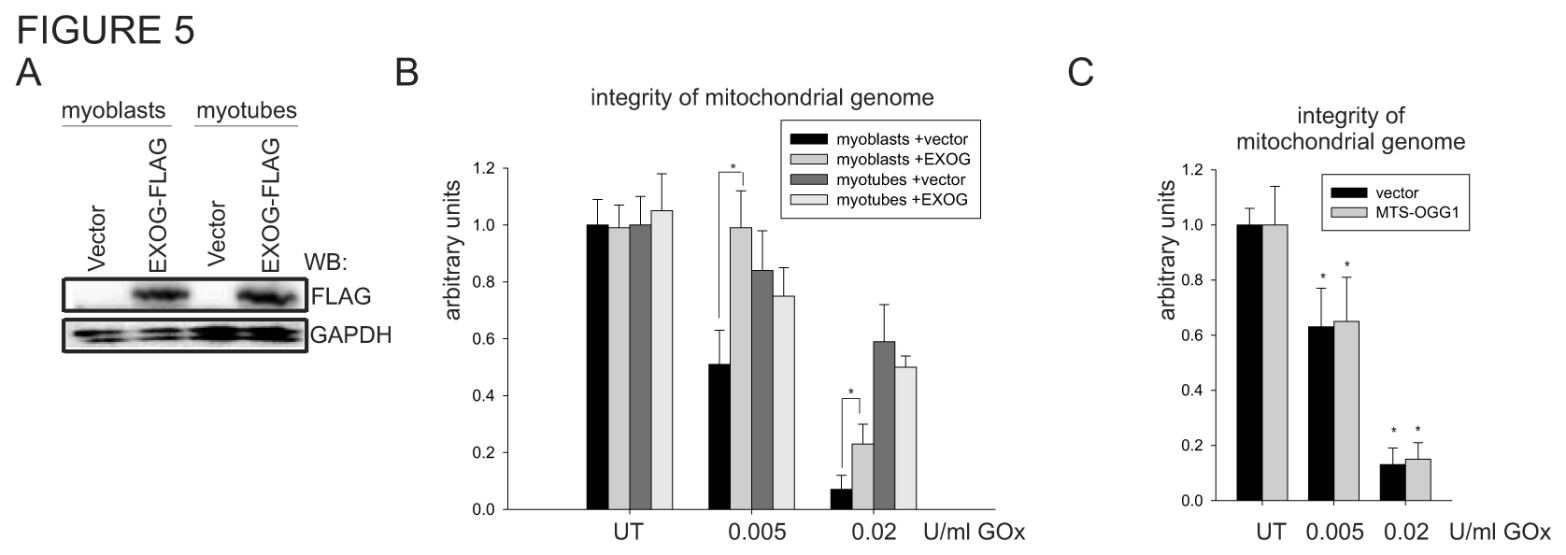
Ectopic expression of EXOG increases resistance to oxidant-induced DNA damage in myoblasts. (**A**) The expression level of EXOG-FLAG-tagged was monitored by Western analysis with FLAG-HRP conjugated antibody. (**B**) The integrity of the mitochondrial genome in myoblasts and myotubes transfected with vector or EXOG expression plasmid after 1 h of treatment with two different concentrations of GOx. The integrity in UT control (empty vector transfected) myoblasts and myotubes was set as 1. (**C**) The integrity of the mitochondrial genome of the myoblasts transfected with vector or mitochondrial specific OGG1 expression plasmid after 1 h of treatment with two different concentrations of GOx. The integrity of the genome was monitored by amplification of the 10kb mitochondrial genome-specific DNA fragment and normalized by mitochondrial genome copy number. The graphs are based on PCR reaction of three independently isolated DNA for each experimental point and shown as mean ± standard error (s.e.m.). Myotubes at day 4 of differentiation were used. * indicates *p* ≤ 0.05. UT, untreated control.

Recently, we characterized a novel 5’ exo/endonuclease, EXOG involved in processing the 5’ end at the DNA strand breaks generated during mitochondrial LP-BER [17,35]. To test our hypothesis that deficiency in 5’ end-processing activities is rate limiting in the repair of the oxidatively damaged mitochondrial genome of myoblasts, we ectopically expressed human EXOG-FLAG-tagged, challenged transfected cells with GOx and monitored the integrity of the mitochondrial genome. Expression of EXOG in both cells was monitored by Western analysis using FLAG-specific antibodies (Figure 5A). A significant resistance to oxidative damage was observed in the EXOG-transfected myoblasts, but not in the myotubes (Figure 5B). As a control, we used overexpression of mitochondria-targeted OGG1 and could not detect any significant changes (Figure 5C). Although several earlier studies showed the protective effect of overexpression of OGG1 in the mitochondria [88,89,90,91], none of these studies investigated its effect on the repair of SSBs in the mitochondrial genome. This observation shows that processing of the 5’ end at the strand breaks in the mitochondrial genome of proliferating myoblasts is the rate limiting step of mitochondrial SSBR, as measured by deficiency in 5’ exonuclease activity provided by EXOG.

### 3.6: Oxidative stress induce apoptosis in myoblasts reduced their viability

**Figure 6:**
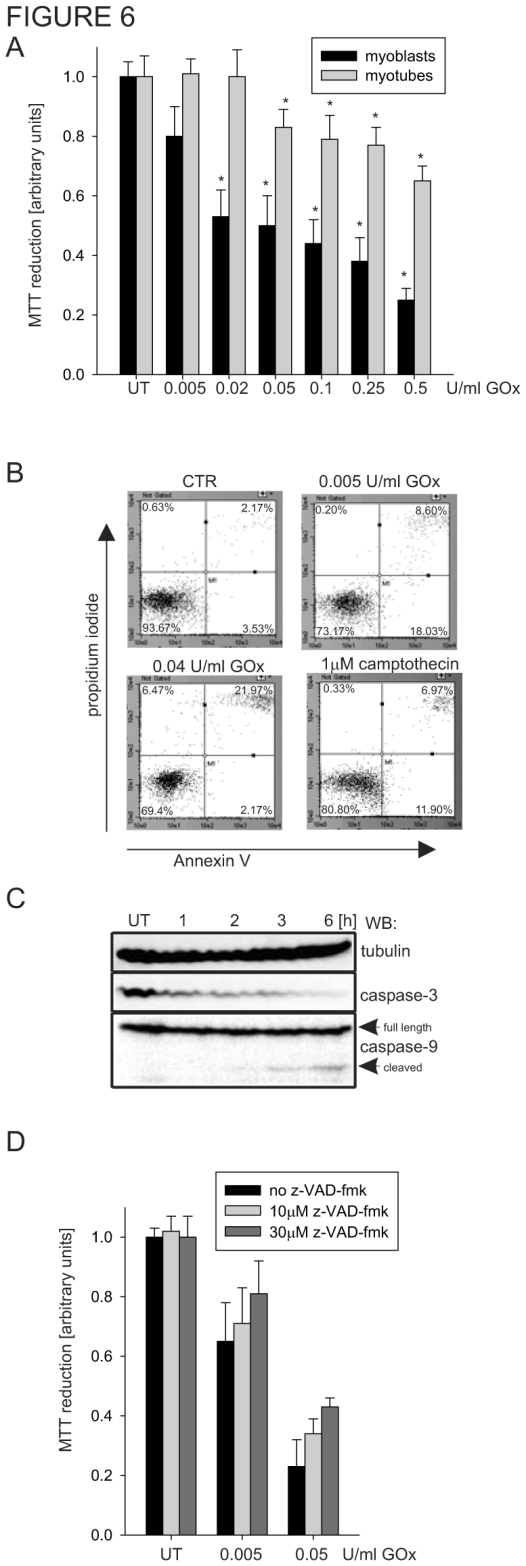
Oxidative stress induces apoptosis in myoblasts. (**A**) Viability of the myoblasts and myotubes upon GOx treatment determined by MTT assay. Viability of untreated, control myoblasts and myotubes was set as 1. (**B**) Flow-cytometry staining using Anexin V/PI of myoblasts 4 h after oxidative challenge. Camptothecin (1 µM) was used as a positive control to induce apoptosis. (**C**) Western analysis of myoblasts treated with 0.01 U GOx/ml at various times. Activation of caspase-9 indicates activation of the intrinsic apoptotic pathway. (**D**) The viability of oxidatively stressed myoblasts was improved by pre-treatment with the pan-specific caspase inhibitor z-VAD-fmk, suggesting inhibition of apoptosis induced by GOx treatment. The graphs are based on three independently experiments and shown as mean ± standard error (s.e.m.). Myotubes at day 4 of differentiation were used. * indicates *p* ≤ 0.05. UT, untreated control.

We have demonstrated so far that oxidants have a more profound negative effect on the integrity of the mitochondrial genomes of myoblasts, but their effect on cellular viability has not yet been explored. We recently showed that accumulation of unrepaired SSBs in the mitochondrial genome alone triggers cell death [17]. Here, we utilized the MTT assay to determine cellular viability in response to oxidative stress [92]. As expected, myoblasts turned out to be more sensitive to GOx treatment than myoblasts, as determined by MTT reduction (Figure 6A). This result strongly supports our previous observation that even a relatively low concentration of GOx (0.02 U/ml) dramatically decreases the integrity of the mitochondrial genome and the viability of myoblasts, but has a marginal effect in myotubes (Figure 2B and Figure 6A). We speculated that, similarly to our prior studies [17], unrepaired mitochondrial genome-specific repair intermediates may activate the intrinsic apoptotic pathway. To explore this possibility, we treated myoblasts with various concentrations of GOx for 1 h and stained the cells with Annexin V/PI, indicating externalization of phosphatidylserine and plasma membrane permeability [93,94]. Cell death was determined 4 h after GOx treatment by flow cytometry. This type of analysis discriminates early apoptotic (Annexin V-positive, PI-negative) from late apoptotic (Annexin V/PI-double positive) or necrotic (Annexin V-negative, PI-positive) cell populations. We detected a significant increase in both early and late apoptotic cell population in lower GOx concentration (Figure 6B). However, higher level of GOx concentration ― in addition to increasing the percentage of apoptotic cells ― also increased the population of Annexin V negative, PI positive cells, indicating necrosis. To further explore the activation of the intrinsic apoptotic pathway, we analyzed the activation of caspase-9 and caspase-3 by Western analysis in myoblasts treated with 0.01 U GOx/ml. As shown in Figure 6C, we detected a decreased signal of uncleaved caspase-3 and of the cleavage product of caspase-9, indicating activation of both caspases. In addition, we tested the effect of the pan-specific caspase inhibitor z-VAD-fmk in the MTT assay. The viability of myoblasts treated with two different concentrations of GOx was significantly higher when cells were pre-treated with z-VAD-fmk (Figure 6D). Together, obtained data showed that unrepaired mitochondrial genome damage initiated cell death, mainly through induction of the apoptotic pathway.

In summary, our results showed that the mitochondrial genome of proliferating C2C12 myoblasts is particularly sensitive to oxidative damage due to a deficiency in the 5’ end-processing at the DNA strand breaks during mitochondrial genome repair via BER/SSBR. We showed that accumulation of repair intermediates, namely SSBs, in the mitochondrial genome of proliferating myoblasts impacts their viability inducing apoptosis. This result supports our previous observation that damage to the mitochondrial genome alone could initiate the intrinsic apoptotic pathway [17]. Significant resistance to the cell death induced by several genotoxic agents in the terminally differentiated muscle cells has been also reported [95,96]. Similarly, resistance to DNA damage was observed in differentiated astrocytes [97]. However, this effect has been linked with a defective signaling pathway of p53 in the myotubes [95,96] or lack of proper DNA damage response due to a deficiency of ATM [97]. The role of mitochondrial genome integrity in these processes has not been studied.

Skeletal muscle represents an important model for studying the role of the mitochondria during cell growth and differentiation. Myoblasts cultured *in vitro* induced by cell confluence and serum deprivation follow a myogenic program including withdrawal from the cell cycle, synthesis of muscle-specific proteins, and fusion onto multinucleated myotubes (Figure 1) [98,99]. Although the importance of the mitochondria in cell growth, cell proliferation, cell death and cell differentiation has been studied previously [100,101], the role of mitochondrial genome damage and its repair during cellular differentiation has not yet been explored. Differentiation appears to be closely associated with mitochondrial function and biogenesis, since increases in mitochondrial mass/volume, mitochondrial DNA copy number, mitochondrial enzyme activity and mitochondrial RNA levels were detected within 48 h of myoblast differentiation (Figure 1 and Figure 4) [100,102]. During aging, satellite cells lose both mitogenic and myogenic abilities, decreasing their number in both mice and humans [103]. We have recently shown that human skeletal muscle satellite cells in older adults are incapable of proliferating when exposed to stimulus, e.g., resistance exercise [104]. Moreover, our unpublished data with human primary skeletal muscle cultures indicate a deficiency in the expression of EXOG in non-Pax7-positevely stain cells, representing the population of replicating stem and progenitor cells (unpublished data). Oxidative stress plays an important role in the development of sarcopenia and in several muscular dystrophies [105,106,107,108]. It has been proposed that accelerated apoptosis in aging muscle may reflect skeletal muscle pathogenesis [108,109]. The results from the present study provide the first clear evidence that proliferating myoblasts are particularly sensitive to oxidative damage due to deficient repair mechanisms in the mitochondrial genome along with lower antioxidant capacities. Our cell culture studies suggest that accumulation of unrepaired damage may be an important factor in the impaired proliferative capabilities of mitochondrial during skeletal muscle regeneration.

## References

[1] Parsons MJ, Green DR (2010) Mitochondria in cell death. Essays Biochem 47: 99-114. doi:10.1042/bse0470099. PubMed: 20533903. 20533903 10.1042/bse0470099

[2] Jeong SY, Seol DW (2008) The role of mitochondria in apoptosis. BMB Rep 41: 11-22. doi:10.5483/BMBRep.2008.41.1.011. PubMed: 18304445. 18304445 10.5483/bmbrep.2008.41.1.011

[3] Kakkar P, Singh BK (2007) Mitochondria: a hub of redox activities and cellular distress control. Mol Cell Biochem 305: 235-253. doi:10.1007/s11010-007-9520-8. PubMed: 17562131. 17562131 10.1007/s11010-007-9520-8

[4] Graier WF, Frieden M, Malli R (2007) Mitochondria and Ca(2+) signaling: old guests, new functions. Pflugers Arch 455: 375-396. doi:10.1007/s00424-007-0296-1. PubMed: 17611770. 17611770 10.1007/s00424-007-0296-1 PMC4864527

[5] Rizzuto R, De Stefani D, Raffaello A, Mammucari C (2012) Mitochondria as sensors and regulators of calcium signalling. Nat Rev Mol Cell Biol 13: 566-578. doi:10.1038/nrm3412. PubMed: 22850819. 22850819 10.1038/nrm3412

[6] Rone MB, Fan J, Papadopoulos V (2009) Cholesterol transport in steroid biosynthesis: role of protein-protein interactions and implications in disease states. Biochim Biophys Acta 1791: 646-658. doi:10.1016/j.bbalip.2009.03.001. PubMed: 19286473. 19286473 10.1016/j.bbalip.2009.03.001 PMC2757135

[7] Richardson DR, Lane DJ, Becker EM, Huang ML, Whitnall M et al. (2010) Mitochondrial iron trafficking and the integration of iron metabolism between the mitochondrion and cytosol. Proc Natl Acad Sci U S A 107: 10775-10782. doi:10.1073/pnas.0912925107. PubMed: 20495089. 20495089 10.1073/pnas.0912925107 PMC2890738

[8] Rouault TA, Tong WH (2005) Iron-sulphur cluster biogenesis and mitochondrial iron homeostasis. Nat Rev Mol Cell Biol 6: 345-351. doi:10.1038/nrg1621. PubMed: 15803140. 15803140 10.1038/nrm1620

[9] Duchen MR, Szabadkai G (2010) Roles of mitochondria in human disease. Essays Biochem 47: 115-137. doi:10.1042/bse0470115. PubMed: 20533904. 20533904 10.1042/bse0470115

[10] Trifunovic A, Wredenberg A, Falkenberg M, Spelbrink JN, Rovio AT et al. (2004) Premature ageing in mice expressing defective mitochondrial DNA polymerase. Nature 429: 417-423. doi:10.1038/nature02517. PubMed: 15164064. 15164064 10.1038/nature02517

[11] Kuzminov A (2001) Single-strand interruptions in replicating chromosomes cause double-strand breaks. Proc Natl Acad Sci U S A 98: 8241-8246. doi:10.1073/pnas.131009198. PubMed: 11459959. 11459959 10.1073/pnas.131009198 PMC37427

[12] Zhou W, Doetsch PW (1993) Effects of abasic sites and DNA single-strand breaks on prokaryotic RNA polymerases. Proc Natl Acad Sci U S A 90: 6601-6605. doi:10.1073/pnas.90.14.6601. PubMed: 8341674. 8341674 10.1073/pnas.90.14.6601 PMC46980

[13] Pesole G, Gissi C, De Chirico A, Saccone C (1999) Nucleotide substitution rate of mammalian mitochondrial genomes. J Mol Evol 48: 427-434. doi:10.1007/PL00006487. PubMed: 10079281. 10079281 10.1007/pl00006487

[14] Kaneko M, Inoue F (1998) The sensitivity to DNA single strand breakage in mitochondria, but not in nuclei, of Chinese hamster V79 and variant cells correlates with their cellular sensitivity to hydrogen peroxide. Toxicol Lett 99: 15-22. doi:10.1016/S0378-4274(98)00132-5. PubMed: 9801026. 9801026 10.1016/s0378-4274(98)00132-5

[15] Mandavilli BS, Santos JH, Van Houten B (2002) Mitochondrial DNA repair and aging. Mutat Res 509: 127-151. doi:10.1016/S0027-5107(02)00220-8. PubMed: 12427535. 12427535 10.1016/s0027-5107(02)00220-8

[16] Vermulst M, Bielas JH, Kujoth GC, Ladiges WC, Rabinovitch PS et al. (2007) Mitochondrial point mutations do not limit the natural lifespan of mice. Nat Genet 39: 540-543. doi:10.1038/ng1988. PubMed: 17334366. 17334366 10.1038/ng1988

[17] Tann AW, Boldogh I, Meiss G, Qian W, Van Houten B et al. (2011) Apoptosis Induced by Persistent Single-strand Breaks in Mitochondrial Genome: CRITICAL ROLE OF EXOG (5'-EXO/ENDONUCLEASE) IN THEIR REPAIR. J Biol Chem 286: 31975-31983. doi:10.1074/jbc.M110.215715. PubMed: 21768646. 21768646 10.1074/jbc.M110.215715 PMC3173182

[18] Almeida KH, Sobol RW (2007) A unified view of base excision repair: lesion-dependent protein complexes regulated by post-translational modification. DNA Repair (Amst) 6: 695-711. doi:10.1016/j.dnarep.2007.01.009. 17337257 10.1016/j.dnarep.2007.01.009 PMC1995033

[19] Fortini P, Dogliotti E (2007) Base damage and single-strand break repair: mechanisms and functional significance of short- and long-patch repair subpathways. DNA Repair (Amst) 6: 398-409. doi:10.1016/j.dnarep.2006.10.008. 17129767 10.1016/j.dnarep.2006.10.008

[20] Hegde ML, Mantha AK, Hazra TK, Bhakat KK, Mitra S et al. (2012) Oxidative genome damage and its repair: implications in aging and neurodegenerative diseases. Mech Ageing Dev 133: 157-168. doi:10.1016/j.mad.2012.01.005. PubMed: 22313689. 22313689 10.1016/j.mad.2012.01.005 PMC3351531

[21] Kazak L, Reyes A, Holt IJ (2012) Minimizing the damage: repair pathways keep mitochondrial DNA intact. Nat Rev Mol Cell Biol 13: 659-671. doi:10.1038/nrm3439. PubMed: 22992591. 22992591 10.1038/nrm3439

[22] Longley MJ, Prasad R, Srivastava DK, Wilson SH, Copeland WC (1998) Identification of 5'-deoxyribose phosphate lyase activity in human DNA polymerase gamma and its role in mitochondrial base excision repair in vitro. Proc Natl Acad Sci U S A 95: 12244-12248. doi:10.1073/pnas.95.21.12244. PubMed: 9770471. 9770471 10.1073/pnas.95.21.12244 PMC22816

[23] Caldecott KW (2008) Single-strand break repair and genetic disease. Nat Rev Genet 9: 619-631. PubMed: 18626472. 18626472 10.1038/nrg2380

[24] Robertson AB, Klungland A, Rognes T, Leiros I (2009) DNA repair in mammalian cells: Base excision repair: the long and short of it. Cell Mol Life Sci 66: 981-993. doi:10.1007/s00018-009-8736-z. PubMed: 19153658. 19153658 10.1007/s00018-009-8736-z PMC11131461

[25] Bolden A, Noy GP, Weissbach A (1977) DNA polymerase of mitochondria is a gamma-polymerase. J Biol Chem 252: 3351-3356. PubMed: 16896. 16896

[26] Pinz KG, Bogenhagen DF (1998) Efficient repair of abasic sites in DNA by mitochondrial enzymes. Mol Cell Biol 18: 1257-1265. PubMed: 9488440. 9488440 10.1128/mcb.18.3.1257 PMC108838

[27] Gao Y, Katyal S, Lee Y, Zhao J, Rehg JE et al. (2011) DNA ligase III is critical for mtDNA integrity but not Xrcc1-mediated nuclear DNA repair. Nature 471: 240-244. doi:10.1038/nature09773. PubMed: 21390131. 21390131 10.1038/nature09773 PMC3079429

[28] Graziewicz MA, Longley MJ, Copeland WC (2006) DNA polymerase gamma in mitochondrial DNA replication and repair. Chem Rev 106: 383-405. doi:10.1021/cr040463d. PubMed: 16464011. 16464011 10.1021/cr040463d

[29] Stierum RH, Dianov GL, Bohr VA (1999) Single-nucleotide patch base excision repair of uracil in DNA by mitochondrial protein extracts. Nucleic Acids Res 27: 3712-3719. doi:10.1093/nar/27.18.3712. PubMed: 10471741. 10471741 10.1093/nar/27.18.3712 PMC148627

[30] Szczesny B, Tann AW, Longley MJ, Copeland WC, Mitra S (2008) Long patch base excision repair in mammalian mitochondrial genomes. J Biol Chem 283: 26349-26356. doi:10.1074/jbc.M803491200. PubMed: 18635552. 18635552 10.1074/jbc.M803491200 PMC2546560

[31] Akbari M, Visnes T, Krokan HE, Otterlei M (2008) Mitochondrial base excision repair of uracil and AP sites takes place by single-nucleotide insertion and long-patch DNA synthesis. DNA Repair (Amst) 7: 605-616. doi:10.1016/j.dnarep.2008.01.002. PubMed: 18295553. 18295553 10.1016/j.dnarep.2008.01.002

[32] Liu P, Qian L, Sung JS, de Souza-Pinto NC, Zheng L et al. (2008) Removal of oxidative DNA damage via FEN1-dependent long-patch base excision repair in human cell mitochondria. Mol Cell Biol 28: 4975-4987. doi:10.1128/MCB.00457-08. PubMed: 18541666. 18541666 10.1128/MCB.00457-08 PMC2519700

[33] Zheng L, Zhou M, Guo Z, Lu H, Qian L et al. (2008) Human DNA2 is a mitochondrial nuclease/helicase for efficient processing of DNA replication and repair intermediates. Mol Cell 32: 325-336. doi:10.1016/j.molcel.2008.09.024. PubMed: 18995831. 18995831 10.1016/j.molcel.2008.09.024 PMC2636562

[34] Cymerman IA, Chung I, Beckmann BM, Bujnicki JM, Meiss G (2008) EXOG, a novel paralog of Endonuclease G in higher eukaryotes. Nucleic Acids Res 36: 1369-1379. PubMed: 18187503. 18187503 10.1093/nar/gkm1169 PMC2275078

[35] Kieper J, Lauber C, Gimadutdinow O, Urbańska A, Cymerman I et al. (2010) Production and characterization of recombinant protein preparations of Endonuclease G-homologs from yeast, C. elegans and humans. Protein Expr Purif 73: 99-106. doi:10.1016/j.pep.2010.04.001. PubMed: 20382228. 20382228 10.1016/j.pep.2010.04.001

[36] Lanza IR, Sreekumaran Nair K (2010) Regulation of skeletal muscle mitochondrial function: genes to proteins. Acta Physiol (Oxf) 199: 529-547. doi:10.1111/j.1748-1716.2010.02124.x. PubMed: 20345409. 20345409 10.1111/j.1748-1716.2010.02124.x PMC3070482

[37] Andreyev AY, Kushnareva YE, Starkov AA (2005) Mitochondrial metabolism of reactive oxygen species. Biochemistry (Mosc) 70: 200-214. doi:10.1007/s10541-005-0102-7. PubMed: 15807660. 15807660 10.1007/s10541-005-0102-7

[38] Szczesny B, Tann AW, Mitra S (2010) Age- and tissue-specific changes in mitochondrial and nuclear DNA base excision repair activity in mice: Susceptibility of skeletal muscles to oxidative injury. Mech Ageing Dev 131: 330-337. doi:10.1016/j.mad.2010.03.009. PubMed: 20363243. 20363243 10.1016/j.mad.2010.03.009 PMC2883317

[39] Richter C, Park JW, Ames BN (1988) Normal oxidative damage to mitochondrial and nuclear DNA is extensive. Proc Natl Acad Sci U S A 85: 6465-6467. doi:10.1073/pnas.85.17.6465. PubMed: 3413108. 3413108 10.1073/pnas.85.17.6465 PMC281993

[40] Yakes FM, Van Houten B (1997) Mitochondrial DNA damage is more extensive and persists longer than nuclear DNA damage in human cells following oxidative stress. Proc Natl Acad Sci U S A 94: 514-519. doi:10.1073/pnas.94.2.514. PubMed: 9012815. 9012815 10.1073/pnas.94.2.514 PMC19544

[41] Anderson S, Bankier AT, Barrell BG, de Bruijn MH, Coulson AR et al. (1981) Sequence and organization of the human mitochondrial genome. Nature 290: 457-465. doi:10.1038/290457a0. PubMed: 7219534. 7219534 10.1038/290457a0

[42] Hiona A, Leeuwenburgh C (2008) The role of mitochondrial DNA mutations in aging and sarcopenia: implications for the mitochondrial vicious cycle theory of aging. Exp Gerontol 43: 24-33. doi:10.1016/j.exger.2007.10.001. PubMed: 17997255. 17997255 10.1016/j.exger.2007.10.001 PMC2225597

[43] Kujoth GC, Hiona A, Pugh TD, Someya S, Panzer K et al. (2005) Mitochondrial DNA mutations, oxidative stress, and apoptosis in mammalian aging. Science 309: 481-484. doi:10.1126/science.1112125. PubMed: 16020738. 16020738 10.1126/science.1112125

[44] Lee CM, Chung SS, Kaczkowski JM, Weindruch R, Aiken JM (1993) Multiple mitochondrial DNA deletions associated with age in skeletal muscle of rhesus monkeys. J Gerontol 48: B201-B205. doi:10.1093/geronj/48.6.B201. PubMed: 8227987. 8227987 10.1093/geronj/48.6.b201

[45] Liu M, Gu L, Sulkin MS, Liu H, Jeong EM et al. (2013) Mitochondrial dysfunction causing cardiac sodium channel downregulation in cardiomyopathy. J Mol Cell Cardiol 54: 25-34. doi:10.1016/j.yjmcc.2012.10.011. PubMed: 23123323. 23123323 10.1016/j.yjmcc.2012.10.011 PMC3595554

[46] Shigenaga MK, Hagen TM, Ames BN (1994) Oxidative damage and mitochondrial decay in aging. Proc Natl Acad Sci U S A 91: 10771-10778. doi:10.1073/pnas.91.23.10771. PubMed: 7971961. 7971961 10.1073/pnas.91.23.10771 PMC45108

[47] Short KR, Bigelow ML, Kahl J, Singh R, Coenen-Schimke J et al. (2005) Decline in skeletal muscle mitochondrial function with aging in humans. Proc Natl Acad Sci U S A 102: 5618-5623. doi:10.1073/pnas.0501559102. PubMed: 15800038. 15800038 10.1073/pnas.0501559102 PMC556267

[48] Figueiredo PA, Mota MP, Appell HJ, Duarte JA (2008) The role of mitochondria in aging of skeletal muscle. Biogerontology 9: 67-84. doi:10.1007/s10522-007-9121-7. PubMed: 18175203. 18175203 10.1007/s10522-007-9121-7

[49] Powers SK, Kavazis AN, McClung JM (2007) Oxidative stress and disuse muscle atrophy. J Appl Physiol 102: 2389-2397. doi:10.1152/japplphysiol.01202.2006. PubMed: 17289908. 17289908 10.1152/japplphysiol.01202.2006

[50] Vasconsuelo A, Milanesi L, Boland R (2008) 17beta-estradiol abrogates apoptosis in murine skeletal muscle cells through estrogen receptors: role of the phosphatidylinositol 3-kinase/Akt pathway. J Endocrinol 196: 385-397. doi:10.1677/JOE-07-0250. PubMed: 18252962. 18252962 10.1677/JOE-07-0250

[51] Sandri M, Carraro U (1999) Apoptosis of skeletal muscles during development and disease. Int J Biochem Cell Biol 31: 1373-1390. doi:10.1016/S1357-2725(99)00063-1. PubMed: 10641792. 10641792 10.1016/s1357-2725(99)00063-1

[52] Dupont-Versteegden EE (2006) Apoptosis in skeletal muscle and its relevance to atrophy. World J Gastroenterol 12: 7463-7466. PubMed: 17167834. 17167834 10.3748/wjg.v12.i46.7463 PMC4087591

[53] Salucci S, Battistelli M, Burattini S, Squillace C, Canonico B et al. (2010) C2C12 myoblast sensitivity to different apoptotic chemical triggers. Micron 41: 966-973. doi:10.1016/j.micron.2010.07.002. PubMed: 20674376. 20674376 10.1016/j.micron.2010.07.002

[54] Frangini M, Franzolin E, Chemello F, Laveder P, Romualdi C et al. (2013) Synthesis of Mitochondrial DNA Precursors during Myogenesis, an Analysis in Purified C2C12 Myotubes. J Biol Chem 288: 5624-5635. doi:10.1074/jbc.M112.441147. PubMed: 23297407. 23297407 10.1074/jbc.M112.441147 PMC3581417

[55] Malinska D, Kudin AP, Bejtka M, Kunz WS (2012) Changes in mitochondrial reactive oxygen species synthesis during differentiation of skeletal muscle cells. Mitochondrion 12: 144-148. doi:10.1016/j.mito.2011.06.015. PubMed: 21782978. 21782978 10.1016/j.mito.2011.06.015

[56] Ramana CV, Boldogh I, Izumi T, Mitra S (1998) Activation of apurinic/apyrimidinic endonuclease in human cells by reactive oxygen species and its correlation with their adaptive response to genotoxicity of free radicals. Proc Natl Acad Sci U S A 95: 5061-5066. doi:10.1073/pnas.95.9.5061. PubMed: 9560228. 9560228 10.1073/pnas.95.9.5061 PMC20213

[57] Nishioka K, Ohtsubo T, Oda H, Fujiwara T, Kang D et al. (1999) Expression and differential intracellular localization of two major forms of human 8-oxoguanine DNA glycosylase encoded by alternatively spliced OGG1 mRNAs. Mol Biol Cell 10: 1637-1652. doi:10.1091/mbc.10.5.1637. PubMed: 10233168. 10233168 10.1091/mbc.10.5.1637 PMC30487

[58] Santos JH, Meyer JN, Mandavilli BS, Van Houten B (2006) Quantitative PCR-based measurement of nuclear and mitochondrial DNA damage and repair in mammalian cells. Methods Mol Biol 314: 183-199. doi:10.1385/1-59259-973-7:183. PubMed: 16673882. 16673882 10.1385/1-59259-973-7:183

[59] Das A, Wiederhold L, Leppard JB, Kedar P, Prasad R et al. (2006) NEIL2-initiated, APE-independent repair of oxidized bases in DNA: Evidence for a repair complex in human cells. DNA Repair (Amst) 5: 1439-1448. doi:10.1016/j.dnarep.2006.07.003. PubMed: 16982218. 16982218 10.1016/j.dnarep.2006.07.003 PMC2805168

[60] Izumi T, Mitra S (1998) Deletion analysis of human AP-endonuclease: minimum sequence required for the endonuclease activity. Carcinogenesis 19: 525-527. doi:10.1093/carcin/19.3.525. PubMed: 9525290. 9525290 10.1093/carcin/19.3.525

[61] Canitrot Y, Lautier D, Laurent G, Fréchet M, Ahmed A et al. (1999) Mutator phenotype of BCR--ABL transfected Ba/F3 cell lines and its association with enhanced expression of DNA polymerase beta. Oncogene 18: 2676-2680. doi:10.1038/sj.onc.1202619. PubMed: 10348341. 10348341 10.1038/sj.onc.1202619

[62] Smirnova E, Toueille M, Markkanen E, Hübscher U (2005) The human checkpoint sensor and alternative DNA clamp Rad9-Rad1-Hus1 modulates the activity of DNA ligase I, a component of the long-patch base excision repair machinery. Biochem J 389: 13-17. doi:10.1042/BJ20050211. PubMed: 15871698. 15871698 10.1042/BJ20050211 PMC1184534

[63] Holloszy JO, Oscai LB, Don IJ, Molé PA (1970) Mitochondrial citric acid cycle and related enzymes: adaptive response to exercise. Biochem Biophys Res Commun 40: 1368-1373. doi:10.1016/0006-291X(70)90017-3. PubMed: 4327015. 4327015 10.1016/0006-291x(70)90017-3

[64] Shen X, Collier JM, Hlaing M, Zhang L, Delshad EH et al. (2003) Genome-wide examination of myoblast cell cycle withdrawal during differentiation. Dev Dyn 226: 128-138. doi:10.1002/dvdy.10200. PubMed: 12508234. 12508234 10.1002/dvdy.10200

[65] Olguin HC, Olwin BB (2004) Pax-7 up-regulation inhibits myogenesis and cell cycle progression in satellite cells: a potential mechanism for self-renewal. Dev Biol 275: 375-388. doi:10.1016/j.ydbio.2004.08.015. PubMed: 15501225. 15501225 10.1016/j.ydbio.2004.08.015 PMC3322464

[66] Moldovan GL, Pfander B, Jentsch S (2007) PCNA, the maestro of the replication fork. Cell 129: 665-679. doi:10.1016/j.cell.2007.05.003. PubMed: 17512402. 17512402 10.1016/j.cell.2007.05.003

[67] Wang YX, Rudnicki MA (2012) Satellite cells, the engines of muscle repair. Nat Rev Mol Cell Biol 13: 127-133. PubMed: 22186952. 10.1038/nrm3265 22186952

[68] Ardite E, Barbera JA, Roca J, Fernández-Checa JC (2004) Glutathione depletion impairs myogenic differentiation of murine skeletal muscle C2C12 cells through sustained NF-kappaB activation. Am J Pathol 165: 719-728. doi:10.1016/S0002-9440(10)63335-4. PubMed: 15331397. 15331397 10.1016/s0002-9440(10)63335-4 PMC1618592

[69] Wagner AE, Ernst IM, Birringer M, Sancak O, Barella L et al. (2012) A combination of lipoic acid plus coenzyme Q10 induces PGC1alpha, a master switch of energy metabolism, improves stress response, and increases cellular glutathione levels in cultured C2C12 skeletal muscle cells. Oxid Med Cell Longev 2012: 835970. 22655115 10.1155/2012/835970 PMC3357652

[70] Ding Y, Choi KJ, Kim JH, Han X, Piao Y et al. (2008) Endogenous hydrogen peroxide regulates glutathione redox via nuclear factor erythroid 2-related factor 2 downstream of phosphatidylinositol 3-kinase during muscle differentiation. Am J Pathol 172: 1529-1541. doi:10.2353/ajpath.2008.070429. PubMed: 18458092. 18458092 10.2353/ajpath.2008.070429 PMC2408414

[71] Kumar S, Sitasawad SL (2009) N-acetylcysteine prevents glucose/glucose oxidase-induced oxidative stress, mitochondrial damage and apoptosis in H9c2 cells. Life Sci 84: 328-336. doi:10.1016/j.lfs.2008.12.016. PubMed: 19159629. 19159629 10.1016/j.lfs.2008.12.016

[72] Hübscher U, Maga G (2011) DNA replication and repair bypass machines. Curr Opin Chem Biol 15: 627-635. doi:10.1016/j.cbpa.2011.08.009. PubMed: 21889903. 21889903 10.1016/j.cbpa.2011.08.009

[73] Pinz KG, Shibutani S, Bogenhagen DF (1995) Action of mitochondrial DNA polymerase gamma at sites of base loss or oxidative damage. J Biol Chem 270: 9202-9206. doi:10.1074/jbc.270.16.9202. PubMed: 7721837. 7721837 10.1074/jbc.270.16.9202

[74] Falkenberg M, Larsson NG, Gustafsson CM (2007) DNA replication and transcription in mammalian mitochondria. Annu Rev Biochem 76: 679-699. doi:10.1146/annurev.biochem.76.060305.152028. PubMed: 17408359. 17408359 10.1146/annurev.biochem.76.060305.152028

[75] Leary SC, Battersby BJ, Hansford RG, Moyes CD (1998) Interactions between bioenergetics and mitochondrial biogenesis. Biochim Biophys Acta 1365: 522-530. doi:10.1016/S0005-2728(98)00105-4. PubMed: 9711303. 9711303 10.1016/s0005-2728(98)00105-4

[76] Lyons CN, Leary SC, Moyes CD (2004) Bioenergetic remodeling during cellular differentiation: changes in cytochrome c oxidase regulation do not affect the metabolic phenotype. Biochem Cell Biol 82: 391-399. doi:10.1139/o04-040. PubMed: 15181473. 15181473 10.1139/o04-040

[77] Siu PM, Wang Y, Alway SE (2009) Apoptotic signaling induced by H2O2-mediated oxidative stress in differentiated C2C12 myotubes. Life Sci 84: 468-481. doi:10.1016/j.lfs.2009.01.014. PubMed: 19302811. 19302811 10.1016/j.lfs.2009.01.014 PMC2778208

[78] Wang Y, Bogenhagen DF (2006) Human mitochondrial DNA nucleoids are linked to protein folding machinery and metabolic enzymes at the mitochondrial inner membrane. J Biol Chem 281: 25791-25802. doi:10.1074/jbc.M604501200. PubMed: 16825194. 16825194 10.1074/jbc.M604501200

[79] Bogenhagen DF, Rousseau D, Burke S (2008) The layered structure of human mitochondrial DNA nucleoids. J Biol Chem 283: 3665-3675. PubMed: 18063578. 18063578 10.1074/jbc.M708444200

[80] Rebelo AP, Dillon LM, Moraes CT (2011) Mitochondrial DNA transcription regulation and nucleoid organization. J Inherit Metab Dis 34: 941-951. doi:10.1007/s10545-011-9330-8. PubMed: 21541724. 21541724 10.1007/s10545-011-9330-8

[81] Furda AM, Marrangoni AM, Lokshin A, Van Houten B (2012) Oxidants and not alkylating agents induce rapid mtDNA loss and mitochondrial dysfunction. DNA Repair (Amst) 11: 684-692. doi:10.1016/j.dnarep.2012.06.002. 22766155 10.1016/j.dnarep.2012.06.002 PMC3878289

[82] Narciso L, Fortini P, Pajalunga D, Franchitto A, Liu P et al. (2007) Terminally differentiated muscle cells are defective in base excision DNA repair and hypersensitive to oxygen injury. Proc Natl Acad Sci U S A 104: 17010-17015. doi:10.1073/pnas.0701743104. PubMed: 17940040. 17940040 10.1073/pnas.0701743104 PMC2040456

[83] Fortini P, Pascucci B, Parlanti E, Sobol RW, Wilson SH et al. (1998) Different DNA polymerases are involved in the short- and long-patch base excision repair in mammalian cells. Biochemistry 37: 3575-3580. doi:10.1021/bi972999h. PubMed: 9530283. 9530283 10.1021/bi972999h

[84] Frosina G, Fortini P, Rossi O, Carrozzino F, Raspaglio G et al. (1996) Two pathways for base excision repair in mammalian cells. J Biol Chem 271: 9573-9578. doi:10.1074/jbc.271.16.9573. PubMed: 8621631. 8621631 10.1074/jbc.271.16.9573

[85] Balakrishnan L, Brandt PD, Lindsey-Boltz LA, Sancar A, Bambara RA (2009) Long patch base excision repair proceeds via coordinated stimulation of the multienzyme DNA repair complex. J Biol Chem 284: 15158-15172. doi:10.1074/jbc.M109.000505. PubMed: 19329425. 19329425 10.1074/jbc.M109.000505 PMC2685697

[86] Liu Y, Beard WA, Shock DD, Prasad R, Hou EW et al. (2005) DNA polymerase beta and flap endonuclease 1 enzymatic specificities sustain DNA synthesis for long patch base excision repair. J Biol Chem 280: 3665-3674. PubMed: 15561706. 15561706 10.1074/jbc.M412922200

[87] Ellenberger T, Tomkinson AE (2008) Eukaryotic DNA ligases: structural and functional insights. Annu Rev Biochem 77: 313-338. doi:10.1146/annurev.biochem.77.061306.123941. PubMed: 18518823. 18518823 10.1146/annurev.biochem.77.061306.123941 PMC2933818

[88] Rachek LI, Thornley NP, Grishko VI, LeDoux SP, Wilson GL (2006) Protection of INS-1 cells from free fatty acid-induced apoptosis by targeting hOGG1 to mitochondria. Diabetes 55: 1022-1028. doi:10.2337/diabetes.55.04.06.db05-0865. PubMed: 16567524. 16567524 10.2337/diabetes.55.04.06.db05-0865

[89] Rachek LI, Grishko VI, Musiyenko SI, Kelley MR, LeDoux SP et al. (2002) Conditional targeting of the DNA repair enzyme hOGG1 into mitochondria. J Biol Chem 277: 44932-44937. doi:10.1074/jbc.M208770200. PubMed: 12244119. 12244119 10.1074/jbc.M208770200

[90] Druzhyna NM, Hollensworth SB, Kelley MR, Wilson GL, Ledoux SP (2003) Targeting human 8-oxoguanine glycosylase to mitochondria of oligodendrocytes protects against menadione-induced oxidative stress. Glia 42: 370-378. doi:10.1002/glia.10230. PubMed: 12730957. 12730957 10.1002/glia.10230

[91] Chatterjee A, Chang X, Nagpal JK, Chang S, Upadhyay S et al. (2008) Targeting human 8-oxoguanine DNA glycosylase to mitochondria protects cells from 2-methoxyestradiol-induced-mitochondria-dependent apoptosis. Oncogene 27: 3710-3720. doi:10.1038/onc.2008.3. PubMed: 18246124. 18246124 10.1038/onc.2008.3

[92] Plumb JA (2004) Cell sensitivity assays: the MTT assay. Methods Mol Med 88: 165-169. PubMed: 14634227. 14634227 10.1385/1-59259-406-9:165

[93] Patel VA, Longacre A, Hsiao K, Fan H, Meng F et al. (2006) Apoptotic cells, at all stages of the death process, trigger characteristic signaling events that are divergent from and dominant over those triggered by necrotic cells: Implications for the delayed clearance model of autoimmunity. J Biol Chem 281: 4663-4670. PubMed: 16377620. 16377620 10.1074/jbc.M508342200 PMC3504611

[94] Büttner S, Eisenberg T, Carmona-Gutierrez D, Ruli D, Knauer H et al. (2007) Endonuclease G regulates budding yeast life and death. Mol Cell 25: 233-246. doi:10.1016/j.molcel.2006.12.021. PubMed: 17244531. 17244531 10.1016/j.molcel.2006.12.021

[95] Fortini P, Ferretti C, Pascucci B, Narciso L, Pajalunga D et al. (2012) DNA damage response by single-strand breaks in terminally differentiated muscle cells and the control of muscle integrity. Cell Death Differ, 19: 1741–9. PubMed: 22705848. 22705848 10.1038/cdd.2012.53 PMC3469061

[96] Latella L, Lukas J, Simone C, Puri PL, Bartek J (2004) Differentiation-induced radioresistance in muscle cells. Mol Cell Biol 24: 6350-6361. doi:10.1128/MCB.24.14.6350-6361.2004. PubMed: 15226436. 15226436 10.1128/MCB.24.14.6350-6361.2004 PMC434249

[97] Schneider L, Fumagalli M, d’Adda di Fagagna F (2012) Terminally differentiated astrocytes lack DNA damage response signaling and are radioresistant but retain DNA repair proficiency. Cell Death Differ 19: 582-591. doi:10.1038/cdd.2011.129. PubMed: 21979466. 21979466 10.1038/cdd.2011.129 PMC3307974

[98] Pownall ME, Gustafsson MK, Emerson CP Jr. (2002) Myogenic regulatory factors and the specification of muscle progenitors in vertebrate embryos. Annu Rev Cell Dev Biol 18: 747-783. doi:10.1146/annurev.cellbio.18.012502.105758. PubMed: 12142270. 12142270 10.1146/annurev.cellbio.18.012502.105758

[99] Sabourin LA, Rudnicki MA (2000) The molecular regulation of myogenesis. Clin Genet 57: 16-25. PubMed: 10733231. 10733231 10.1034/j.1399-0004.2000.570103.x

[100] Kraft CS, LeMoine CM, Lyons CN, Michaud D, Mueller CR et al. (2006) Control of mitochondrial biogenesis during myogenesis. Am J Physiol Cell Physiol 290: C1119-C1127. PubMed: 16531567. 16531567 10.1152/ajpcell.00463.2005

[101] Duguez S, Sabido O, Freyssenet D (2004) Mitochondrial-dependent regulation of myoblast proliferation. Exp Cell Res 299: 27-35. doi:10.1016/j.yexcr.2004.05.017. PubMed: 15302570. 15302570 10.1016/j.yexcr.2004.05.017

[102] Moyes CD, Mathieu-Costello OA, Tsuchiya N, Filburn C, Hansford RG (1997) Mitochondrial biogenesis during cellular differentiation. Am J Physiol 272: C1345-C1351. PubMed: 9142861. 9142861 10.1152/ajpcell.1997.272.4.C1345

[103] Gopinath SD, Rando TA (2008) Stem cell review series: aging of the skeletal muscle stem cell niche. Aging Cell 7: 590-598. doi:10.1111/j.1474-9726.2008.00399.x. PubMed: 18462272. 18462272 10.1111/j.1474-9726.2008.00399.x

[104] Walker DK, Fry CS, Drummond MJ, Dickinson JM, Timmerman KL et al. (2012) PAX7+ satellite cells in young and older adults following resistance exercise. Muscle Nerve 46: 51-59. doi:10.1002/mus.23266. PubMed: 22644638. 22644638 10.1002/mus.23266 PMC3374905

[105] Jackson MJ, McArdle A (2011) Age-related changes in skeletal muscle reactive oxygen species generation and adaptive responses to reactive oxygen species. J Physiol 589: 2139-2145. doi:10.1113/jphysiol.2011.206623. PubMed: 21320885. 21320885 10.1113/jphysiol.2011.206623 PMC3098693

[106] Brown RH (1995) Free radicals, programmed cell death and muscular dystrophy. Curr Opin Neurol 8: 373-378. doi:10.1097/00019052-199510000-00009. PubMed: 8542043. 8542043 10.1097/00019052-199510000-00009

[107] Marzetti E, Leeuwenburgh C (2006) Skeletal muscle apoptosis, sarcopenia and frailty at old age. Exp Gerontol 41: 1234-1238. doi:10.1016/j.exger.2006.08.011. PubMed: 17052879. 17052879 10.1016/j.exger.2006.08.011

[108] Marzetti E, Calvani R, Bernabei R, Leeuwenburgh C (2011) Apoptosis in skeletal myocytes: a potential target for interventions against sarcopenia and physical frailty - a mini-review. Gerontology 58: 99-106. PubMed: 21952604. 21952604 10.1159/000330064 PMC7077073

[109] Marzetti E, Hwang JC, Lees HA, Wohlgemuth SE, Dupont-Versteegden EE et al. (2010) Mitochondrial death effectors: relevance to sarcopenia and disuse muscle atrophy. Biochim Biophys Acta 1800: 235-244. doi:10.1016/j.bbagen.2009.05.007. PubMed: 19450666. 19450666 10.1016/j.bbagen.2009.05.007 PMC2826514

